# AcroVoice: eliciting the patients’ perspective on acromegaly disease activity

**DOI:** 10.1007/s11102-018-00933-9

**Published:** 2019-01-09

**Authors:** Yanina Jackson, Emuella Flood, Stephanie Rhoten, Ellen M. Janssen, Mark Lundie

**Affiliations:** 1 Pfizer Inc, Rare Disease Medical Affairs, New York, NY USA; 2 Patient Centered Outcomes, ICON, Gaithersburg, MD USA; 3 Patient Centered Outcomes, ICON, South San Francisco, CA USA; 4 Pfizer Canada Inc, Rare Disease Medical Affairs, Kirkland, QC Canada

**Keywords:** Acromegaly, Disease management, Patient-centric, Quality of life, Patient preferences, Discrete choice experiment

## Abstract

**Purpose:**

To determine how patients define acromegaly disease activity and treatment success and to quantify from the patients’ perspective the relative importance of each disease parameter included in the ACRODAT®.

**Methods:**

One hundred acromegaly patients on medical therapy (mean age = 47.1 years; SD = 11.96) completed an online preference study evaluating hypothetical patient profiles described in terms of insulin-like growth factor-I (IGF-I) levels, tumor size, comorbid conditions, signs/symptoms, and quality of life (QoL). Participants first completed a single-profile task experiment by rating 20 single patient profiles as exhibiting stable, mild, or significant disease activity based on treatment success. Next, participants completed a double-profile discrete choice experiment (DCE) by selecting the patient that was doing “better” from 15 profile pairs. Results were analyzed using logistic and conditional logistic models.

**Results:**

When choosing between stable vs. mild or significant disease activity, signs/symptoms, tumor size, and IGF-I levels were weighted equally; IGF-I and signs and symptoms were valued equally when selecting mild vs. significant disease activity. The DCE showed that, statistically, all disease parameters, except comorbid conditions, predicted health status equally. Tumor size and IGF-I levels each accounted for 23% of the decision-making process; QoL, signs/symptoms, and comorbid conditions accounted for 21%, 19%, and 14%, respectively.

**Conclusion:**

All five ACRODAT® parameters had some influence on disease activity from the patients’ perspective. To account for patients’ preferences and optimize treatment and outcomes, a holistic disease management approach should be employed.

**Electronic supplementary material:**

The online version of this article (10.1007/s11102-018-00933-9) contains supplementary material, which is available to authorized users.

## Introduction

Acromegaly is a rare progressive condition which occurs in approximately 60 per 1 million people, and is primarily caused by excess growth hormone (GH) secreted by a benign pituitary adenoma [1, 2]. The condition is characterized by enlarged extremities (e.g., hands, feet, jaw, and facial bones) and soft tissue growth, and is associated with significant morbidity and mortality. The most serious health consequences of acromegaly include type 2 diabetes, high blood pressure, increased risk of cerebrovascular and cardiovascular disease, and arthritis [3, 4]. If left untreated or if treated sub-optimally, acromegaly can lead to serious illness and premature death [5]. Although symptoms can appear at any age, acromegaly is most often diagnosed in middle-aged adults, and diagnosis is often delayed due to its low prevalence and the insidious onset of signs/symptoms [6].

Treatment for acromegaly primarily involves surgical resection of the tumor followed by pharmacotherapy or radiation to maintain hormone homeostasis [7]. Traditional clinical treatment goals include biochemical control (i.e., normalization of GH and insulin-like growth factor I; IGF-I), tumor size reduction, as well as improved signs/symptoms [7, 8]. While biochemical control is the main goal of medical therapy, even when it is achieved, many patients continue to experience symptoms and impaired health-related quality of life (QoL) [9, 10].

Most community-based endocrinologists and primary care physicians treat very few cases of acromegaly during their practice. To aid in clinical management of acromegaly and to support a holistic approach to patient care, a multi-dimensional clinical decision support tool, the ACRODAT® (Acromegaly Disease Activity Tool) [10], was developed to help predict what an expert endocrinologist would consider as stable (S), mild (M-DA), or significant disease activity (S-DA). The ACRODAT® evaluates five disease parameters, including two “clinical” parameters (IGF-I levels and tumor status) and three “patient-centered” parameters (comorbid conditions, signs/symptoms, and QoL). A physician validation study to inform ACRODAT® development showed that expert endocrinologists mainly valued the two “clinical” parameters in their assessment of disease activity, while their assessment was minimally influenced by the remaining, more patient-oriented parameters [10]. However, it is unclear how patients with acromegaly assess the relative importance of these five disease parameters, and if their perspectives differ from those of physicians.

Examining patient preferences can provide valuable insight into the relative importance of treatment attributes from the patients’ point of view, and the trade-offs that patients are willing to make with respect to efficacy, tolerability, and convenience of a particular treatment modality [11]. Patient preference studies can also be used to identify the type and level of side effects that patients are willing to tolerate for a given treatment benefit [11, 12]. To further support a holistic approach to acromegaly disease management, this study examined patients’ perspectives on acromegaly disease activity by identifying which disease parameters are important to patients for defining disease activity and treatment success, and quantifying the relative importance of each disease parameter included in the ACRODAT® on overall disease activity.

## Methods

To assess the relative importance of acromegaly disease parameters on overall disease activity and treatment decision making from the patient perspective, this study utilized a choice-based methodology with a quantitative patient preference survey developed following qualitative interviews with patients. Ethical approval for the study was received in Canada and the United States.

### Survey development

#### Concept elicitation interviews

One-time, semi-structured, qualitative concept elicitation (CE) telephone interviews (60–90 min) were conducted with seven post-surgical adult patients with acromegaly to explore the factors they considered to be important in evaluating their disease activity, whether the five disease parameters included in ACRODAT® could reasonably be included in a patient preference survey, and if any additional disease-related factors are important to patients [13]. Patients identified similar parameters as those clinicians identified for inclusion in the ACRODAT®, including signs/symptoms, IGF-I levels, comorbid conditions, and QoL. Patients viewed “numbers” (i.e., IGF-I levels) as important indicators of disease activity; however, patients also indicated that while physicians tend to focus on the “numbers,” these do not fully capture their experience of living with acromegaly. Tumor status was also important to patients when describing acromegaly, though this was generally elicited only after probing. In terms of treatment outcomes, patients primarily considered changes in symptoms and biochemical test results as indicators of treatment effectiveness. This qualitative study concluded that the five health parameters included in the ACRODAT® were appropriate to include in a patient preference survey [13].

#### Choice tasks development

The quantitative patient preference survey included the five ACRODAT® parameters. Each parameter was described across three severity levels (Table 1), from least (level 1) to most severe (level 3). The description of comorbid conditions was modified and phrased as “related conditions” in the survey to aid patient interpretation. To assess the relative importance of parameters to patients for treatment decision making and overall health assessment, two separate sets of preference elicitation tasks were constructed, including single-profile and double-profile discrete choice experiment (DCE) choice tasks, respectively.

**Table 1:** Parameters and levels

Parameter	Parameter levels with definitions		
	Level 1 (least severe)	Level 2 (moderately severe)	Level 3 (most severe)
IGF-I levels	IGF-I levels are within normal limits	Slightly elevated above the normal range	Significantly elevated above the normal range
Tumor size	No change: not visible or has not changed since the previous MRI	Slight increase in size	Significant increase in size or has spread
Signs and symptoms^a^	None or mild	Moderate acromegaly symptoms	Severe acromegaly symptoms
Comorbid conditions^a^	None or mild: does not have diabetes or sleep apnea OR Has cardiac disease, but currently is well controlled	Moderate: has currently controlled diabetes and no cardiac disease or sleep apnea OR Has moderate sleep apnea OR Has cardiac disease that is not well controlled	Severe: poorly controlled diabetes OR Well controlled diabetes, but cardiovascular disease in poorly controlled and signs of moderate to severe sleep apnea
Quality of life	No or minimal impairment in QoL	Moderate impairment in QoL	Significant impairment in QoL

*IGF-I* insulin-like growth factor I, *MRI* magnetic resonance imaging, *QoL* quality of life

^a^The survey used patient-friendly language including describing “comorbid conditions” as “related conditions” and “signs and symptoms” simply as “symptoms”

##### Single-profile choice tasks

The single-profile choice tasks assessed how patients evaluate the five disease parameters when deciding if a change in acromegaly treatment is needed. Each participant was shown 20 profiles and were asked to select whether they believed: (1) the patient is doing well and a change in treatment is not required (i.e. stable; S); (2) the patient should consider seeing a doctor as a change in treatment might be needed (i.e. mild disease activity; M-DA), or (3) the patient needs to see a doctor as a change in treatment is required (i.e. severe disease activity; S-DA). The order of the profiles and of health parameters in the profiles was randomized across participants to minimize order bias [14].

##### Double-profile DCE choice tasks

The double-profile DCE choice tasks assessed how patients weigh the five disease parameters when quantifying overall acromegaly disease activity. In this set of tasks, participants were shown two full patient profiles (Patient A and Patient B) in random order [14] and asked to select the patient who they believed was doing better.

To select the profiles in each double-profile DCE choice task, an iterative experimental design was used in which the profiles were updated twice to account for prior patients’ responses [15]. The iterative approach was taken in an attempt to increase the statistical efficiency of the subsequent iteration, and potentially reduce the sample size required to achieve statistically significant results [16]. All designs were constructed in NGene [17] and made use of a D-efficient design strategy to construct 15 full-profile, paired DCE tasks [18].

### Preference survey recruitment

Survey participants were recruited from four patient organizations in the United States (n = 69) and Canada (n = 31). Three of the patient organizations were in geographically diverse areas of Canada and one from the United States. Participants provided electronic informed consent and were required to have a self-reported physician diagnosis of acromegaly, be post-surgical or ineligible for surgery, and have been taking acromegaly medication for at least a year.

### Statistical analysis

#### Single-profile choice tasks

The single-profile choice task results were examined using logistic regression models that were similar to the approach in the physician study [10]. The logistic regression models examined the relationship between severity assessment and the different disease parameters. The analysis modeled the participants’ choices as two separate binary choices: the choice between S versus M-DA/S-DA and the choice between M-DA versus SDA.

Disease parameter coefficients resulting from the models were converted into odds ratios (ORs) for ease of interpretation. An OR above 1 indicates that the disease parameter level was positively associated, or had greater odds, with choosing more severe disease classification. Wald tests were also conducted comparing the ORs for each disease parameter level to the OR for the corresponding IGF-I parameter level. These comparisons between ORs were used to determine if the IGF-I levels parameter had a reliably similar influence on participants’ treatment-seeking decision compared to the other disease parameters. The IGF-I levels parameter was chosen as the comparator because it is commonly used in clinical practice to assess disease activity and was also found to be the most important parameter in the physician study [10].

#### Double-profile DCE choice tasks

The choices participants made in the double-profile DCE were analyzed using a conditional logistic model and dummy coding was used for all variables [19]. Similar to the analysis of the single-profile choice tasks, in the conditional logistic model, the choice on the patient profile was the outcome variable and the disease parameter levels were the independent variables.

To help interpret the results of the double-profile DCE, the relative importance of each disease parameter was calculated by first determining the coefficient difference. To estimate the relative importance of a single attribute as a percentage, the coefficient difference for that attribute was then divided by the sum of the coefficient differences for all of the attributes.

## Results

### Survey participants

One hundred participants with acromegaly (65 females and 35 males) took part in the study, including 69 from the United States and 31 from Canada. Participants were diagnosed with acromegaly between 1.27 and 37.45 years ago (mean = 10.35; SD = 6.83), and their current self-reported acromegaly treatments included somatostatin analogues (SSAs; 64%), pegvisomant (32%), dopamine agonists (13%), and radiation therapy (6%). Participants’ additional demographic and socioeconomic characteristics are shown in Table 2.

**Table 2:** Demographic and socioeconomic characteristics of analysis population (n = 100)

Characteristic	n	%
Age (years), Mean (SD)	47.71 (11.96)	
Sex		
Female	65	65
Race/ethnicity^a^		
White	92	92
Black or African American (US only)	3	3
Asian (US only)	3	3
Asian or Pacific Islander (CA only)	1	1
American Indian or Alaska Native (US only)	1	1
Hispanic or Latino	4	4
Other	2	2
Country		
Canada	31	31
United States	69	69
Highest level of education completed		
Did not complete high school	4	4
High school diploma or equivalent (GED)	15	15
Some college	22	22
2-year diploma	7	7
4-year bachelor’s degree	28	28
Master’s degree	9	9
Doctoral or professional degree	11	11
Other	4	4
Acromegaly duration in years (since diagnosis), Mean (SD)	10.35 (6.83)	
Endocrinologist office location		
Major hospital or university	66	66
Community hospital or clinic	16	16
Private practice	18	18
Current medication/treatment^ab^		
Somatostatin analogues/SSAs	64	64
Pegvisomant	32	32
Dopamine agonists	13	13
Radiation	6	6
Previous medication/treatment^a^		
Somatostatin analogues/SSAs	45	45
Pegvisomant	27	27
Dopamine agonist	36	36
Radiation	27	27

^a^Multiple responses allowed, percentages do not add to 100

^b^Open-ended question in which acromegaly specific medications were included

#### Single-profile tasks

Some preference heterogeneity was observed between participants in the single-profile choices. In the first binary choice (between S and M-DA/S-DA), the frequency with which participants classified a profile as M-DA/S-DA ranged from 74.7% for normal IGF-I levels to 95.6% for significant changes in tumor size. In the second binary choice (between M-DA and S-DA), the frequency with which a profile was rated as S-DA ranged from 38.5% for no changes in tumor size to 71.3% for significant changes in tumors size.

**Fig. 1:**
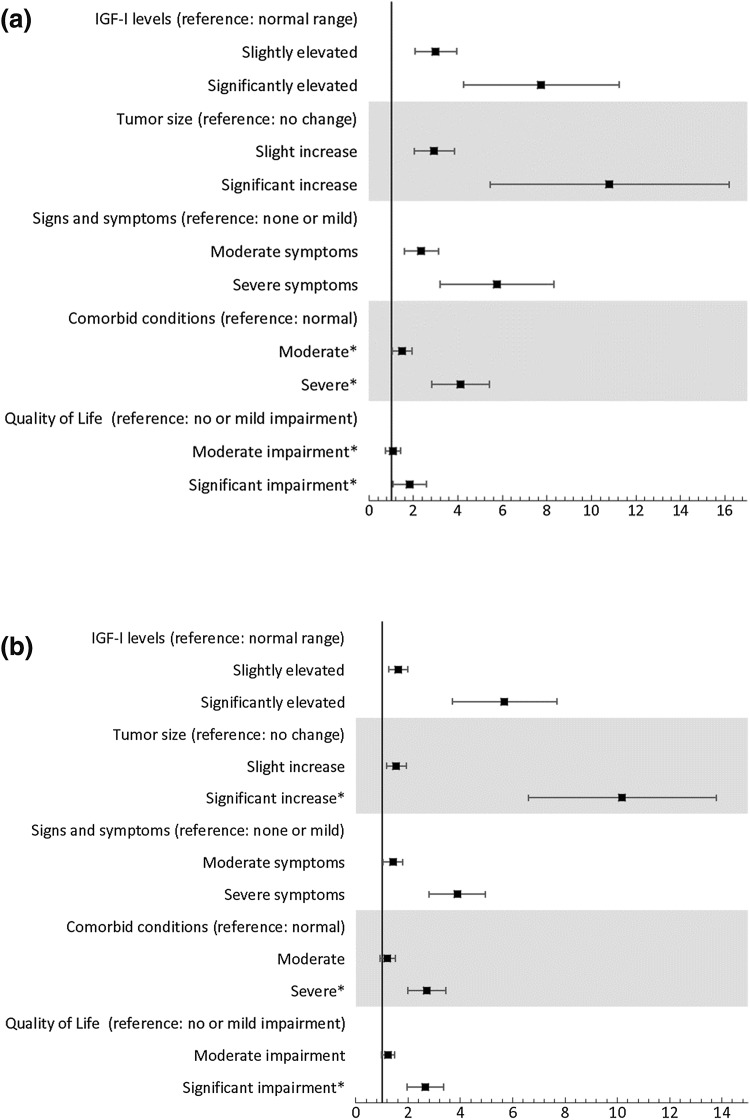
Single-profile choice task regression results: **a** predicting participant assessments of mild disease activity or significant disease activity (compared to stable); and **b** predicting participant assessments of significant disease activity (compared to mild disease activity). Odd Ratios (ORs) with 95% confidence interval (CI) are shown on the horizontal axes. Note: An OR above 1 indicates that participants were more likely to choose more severe disease activity when the parameter level of interest was shown. In this figure, the blocks represent the mean OR, and the brackets span the 95% Confidence interval. A disease parameter level has a significant impact on choices if the 95% CI does not cross zero. ^a^The survey used patient-friendly language which described “comorbid conditions” as “related conditions” and “signs and symptoms” as “symptoms”. *OR significantly different from the IGF-I disease parameter for the same attribute level

In the first binary choice (between S and M-DA/S-DA), results suggests that participants were equally influenced by acromegaly signs/symptoms and tumor size levels as by IGF-I levels, but that comorbid conditions and QoL were less influential than IGF-I levels (Fig. 1a). No statistically significant differences were found when comparing IGF-I levels to tumor size and signs/symptoms (p > 0.05). However, the ORs for comorbid conditions and QoL were significantly less than the ORs for IGF-I (p < 0.05). Overall, patient profiles were more likely to be indicated as M-DA/S-DA instead of S when they contained more severe disease parameter levels. IGF-I levels, tumor size, and signs/symptoms carried the most weight, followed by QoL and comorbid conditions. All ORs were significantly greater than zero, except for moderate levels of comorbid conditions, indicating that each parameter was used, although not at every level, to differentiate between S and M-DA/S-DA.

In the second binary choice (between M-DA and S-DA), IGF-I levels and signs/symptoms were equally influential both at the moderate and severe levels (p > 0.05) (Fig. 1b). Compared to moderate IGF-I levels, the moderate levels of tumor size, comorbid conditions, and QoL also carried the same weight when choosing between M-DA and S-DA (p > 0.05). However, significant changes in tumor size was statistically more important than IGF-I levels (p < 0.05), while severe comorbid conditions and significant impairment in QoL carried less weight in this choice (p < 0.05). Patient profiles were more likely to be classified as S-DA instead of M-DA when they contained more severe disease parameter levels. In the choice between S-DA and M-DA, tumor size carried the most weight, followed by IGF-I levels, signs/symptoms, comorbid conditions, and QoL. All ORs, except for mild to moderate impairment in QoL and moderate levels of comorbid conditions, were significantly larger than zero, indicating that all disease parameter levels, except for the ones noted, were used as part of participants’ decision to differentiate S-DA and M-DA patient profiles.

### Double-profile DCE

Some preference heterogeneity was observed in the double-profile DCE tasks; for none of the tasks was one treatment profile chosen as representing better health status by all participants. The largest difference was for a profile pair in which 74% of participants chose Profile 1 as having the most preferable health status and 26% chose Profile 2.

**Fig. 2:**
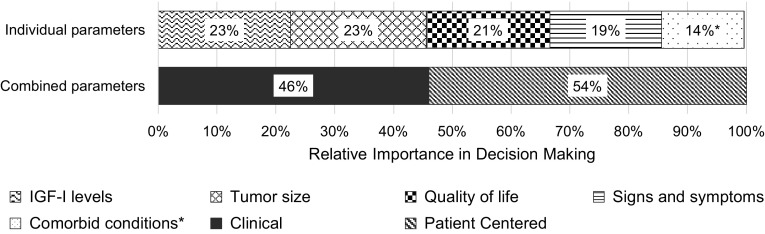
Relative importance of disease parameters on overall health status from the double-profile DCE for each parameter individually and grouped by parameter type. Note: Clinical parameters include IGF-I levels and tumor change; patient-centered parameters include quality of life signs and symptoms, and comorbid conditions. *relative importance of comorbid conditions statistically different from the relative importance of IGF-I levels

In the double-profile DCE, all disease parameters (p > 0.05), except for comorbid conditions (p < 0.01), were statistically similar to IGF-I levels. Tumor size and IGF-I levels were the most highly valued parameters, each accounting for 23% of the decision-making process. QoL, signs/symptoms, and comorbid conditions accounted for 21%, 19%, and 14% of the decision-making process, respectively. The combined importance of the two “clinical” disease parameters (tumor status and IGF-I levels) was 46%, and the combined importance of the three “patient-centered” parameters (signs/symptoms, QoL, and comorbid conditions), was 54% (Fig. 2).

## Discussion

In this study, we identified which disease parameters patients use to define their acromegaly disease activity in terms of health status (double profile DCE choice tasks) and changing treatment needs (single-profile choice task) as well as estimated the relative importance/predictive value of the five acromegaly disease parameters included in the ACRODAT® on overall disease activity from the patients’ perspective. Results showed that all five acromegaly disease parameters included in the ACRODAT® were important to patients for assessing acromegaly disease activity. Our results indicate that patient-centered parameters (i.e., signs/symptoms) as well as other clinical parameters were strongly considered when making choices about treatment decisions.

Both in the single and double profile tasks, comorbid conditions had less influence on patients’ choices than other parameters. Patients may have placed less importance on comorbid conditions due to limited understanding of the difference between symptoms and comorbid conditions, a finding supported by the qualitative interviews [13]. This highlights the need for more patient-targeted education to ensure that patients and physicians use similar terms, and to enable shared decision-making and a more holistic approach to disease management.

The QoL parameter was highly influential when patients selected their preferred health status (double-profile DCE), but was ranked as the least important disease parameter when evaluating the need for treatment changes (single-profile choice tasks). This suggests that QoL may better predict patients’ assessment of their health status than their decisions surrounding treatment changes. Patients might assess differences in clinical factors used by their physicians, such as IGF-I levels and tumor size, to seek changes in treatment, but assess differences in patient-centered concepts, such as QoL, when they consider their overall well-being. These findings are once again supported by the qualitative results, in which patients described IGF-I levels as “numbers” used by their clinicians, and used the patient-centered parameters, such as symptoms and QoL to discuss their overall health [13].

In contrast to findings from the physician validation study [10], patients placed more importance on patient-centered parameters (i.e., signs/symptoms, comorbid conditions, and QoL) than did expert endocrinologists. The clinical parameters dominated the physician’s assessment of disease activity, and the three patient-centered parameters only influenced physicians’ assessments of disease activity if IGF-I levels and tumor size were not present at the highest level of severity [10]. In contrast, for patients, the patient-centered parameters in this study were as important as the “clinical” parameters. In the double-profile DCE tasks, patients even valued the three patient-centered parameters combined more than the two “clinical” parameters combined.

As patients are key experts on their health and disease, optimum patient care should account for patient preferences, and shared decision-making tools should incorporate both physicians’ and patients’ perspectives. As such, input from patients and caregivers, in addition to input from healthcare providers, is a best practice for clinical disease management and associated tool development. This is especially important in a rare disease setting in which, with the exception of a limited number of experts, healthcare providers are not as familiar with a particular condition and might rely more heavily on clinical decision-making tools to support disease management. In these settings, patient advocacy and support groups can also play an important role in educating and empowering patients to actively participate in their own care.

Equally important is the inclusion of the patients’ voice in research. In this study, we had the privilege of working directly with patient support groups, which, given the rarity of acromegaly, led to the recruitment of a relatively large sample of participants. This would not have been possible without the direct involvement and engagement of the patient support group members and leadership. Partnerships between researchers and patient groups are especially important for rare diseases to ensure that the patient perspective is heard and incorporated [20, 21].

This study had a few limitations. First, patients were asked to make hypothetical choices for which they did not face consequences, which might impede real world generalizability of the results. For example, in the single-profile experiment, few patients identified the profile patient as “stable”, regardless of the parameter levels presented. However, even though participants indicated that a patient should consider or request a change in treatment, it is unlikely that acromegaly patients are frequently requesting a change in treatment in the real world. Second, given that acromegaly is a rare disease, we were only able to survey a relatively small group of patients when considering standard practices for preference research [22, 23]. Given that preference elicitation methods might be especially valuable in rare disease populations for which clinical trial data is limited, it is important to implement strategies that enable meaningful analysis of data collected from small sample sizes, which are expected in rare disease research. In this study we employed an efficient study design by using an iterative approach to update the experimental design. This, along with other strategies, such as asking participants to complete more choice tasks over multiple sittings, should be explored to overcome study size limitations. Third, for the single-profile choice tasks, two binary logistic regression models were used rather than a cumulative logistic regression to more closely approximate real-world clinical decisions making. No modification to the analysis was made to reflect that the two different regressions modeled related concepts with the same data. Our presented results might therefore slightly overestimate the ORs for the M-DA versus S-DA choice. This choice was made to keep the analytical approach similar to the previously conducted clinician study [10]. Finally, numerous p-values were calculated to make comparisons within and across disease parameters. This multiplicity, uncontrolled for type-I error, can increase the chances of finding “false positive” statistically significant results.

## Conclusion

In this patient preference study, we found that for patients with acromegaly, all five ACRODAT® parameters influence some choices around disease activity. Both “clinical” (tumor size and IGF-I level) and “patient-centered” parameters (signs/symptoms, comorbid conditions, and QoL) influenced how patients define acromegaly disease activity, either in terms of overall health status or the need to change treatment. In contrast with the physician validation study which favored “clinical” parameters, patients placed more value than expert endocrinologists on the “patient-centered” parameters and often weighted them equally. These findings suggest that patient-centric disease parameters play an important role in shaping patients’ preferences surrounding their health status and treatment decision-making. Furthermore, while patients may not spontaneously describe their own disease in terms of IGF-I levels and/or tumor size, they do appreciate the importance of these factors in evaluating acromegaly disease activity. It is important to make clinicians aware of these findings to ensure that they take a holistic approach to clinical management of acromegaly that is in line with patient preferences.

## Electronic supplementary material

Below is the link to the electronic supplementary material.

Supplementary material 1 (PDF 591 KB)
