# Supplementary material for: AcroVoice: eliciting the patients’ perspective on acromegaly disease activity
Source: Pituitary. 2019 Jan 9;22(1):62–9. doi: 10.1007/s11102-018-00933-9 (PMC6373299; doi:10.1007/s11102-018-00933-9)
Supplement: Supplementary file 1 — Supplementary material 1 (PDF 591 KB) [file 11102_2018_933_MOESM1_ESM.pdf]

Appendices

Appendix A: Sample choice tasks

- Below is a patient profile with descriptions for each of the 5 characteristics.
- Take your time and review the patient's profile carefully. Choose by clicking the appropriate button, depending on whether you believe:

A) The patient is doing well and a change in treatment is not required,

B) The patient should consider seeing a doctor as a change in treatment might be needed, or

C) The patient needs to see a doctor as a change in treatment is required.

Health-Related Characteristic	Patient Description
IGF-1 Levels	Slightly elevated above normal range
Related Conditions	Normal
Symptoms	Moderate
Quality of Life	No or minimal impairment
Tumor Size	Slight increase in size

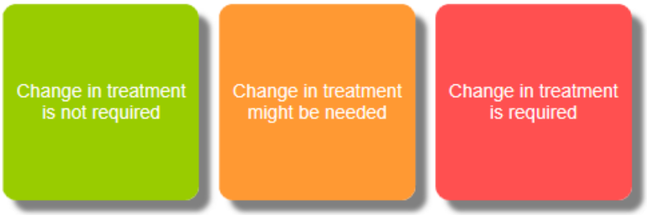

Figure A-1 Example of Single-profile choice task

- Below are two patient profiles with different characteristics. Please assume that everything else about the patients is exactly the same.
- Take your time and review each profile carefully. Choose the patient that you believe is doing **better** by clicking the button at the bottom of the screen under Patient A or Patient B.

Characteristic	Patient A	Patient B
IGF-1 Levels	Significantly elevated above normal range	Within normal limits
Related Conditions	Moderate	Normal
Symptoms	Severe	None or mild
Quality of Life	Moderate impairment	Significant impairment
Tumor Size	Slight increase in size	Significant increase in size or has spread
Which patient is doing **better**?	○	○

Figure A-2 Example of a Double-profile DCE task

Appendix B: Single-profile and double-profile DCE results tables

Table B-1 Single-profile choice task overall frequency and output of multivariable model assessing choices for [Stable] vs [Mild Disease Activity or Significant Disease Activity]

Disease parameter	Participant Assessment of presence of disease activity: Stable vs any disease Activity (M-DA or S-DA)				
	Frequency of selection		Multivariable model (Stable is reference outcome)		
	Stable	M-DA or S-DA	OR	95% CI	P-value
IGF-I					
Normal	25.3% (152)	74.7% (448)	(Ref)	(Ref)	(Ref)
Slightly elevated	9.3% (70)	90.7% (680)	3.00	(2.07, 4.35)	<.0001
Significantly elevated	6.5% (42)	93.5% (608)	7.74	(4.24, 14.14)	<.0001
Tumor size					
No change	24.5% (159)	75.5% (491)	(Ref)	(Ref)	(Ref)
Slight increase	12.0% (72)	88.0% (528)	2.94	(2.04, 4.23)	<.0001
Significant increase	4.4% (33)	95.6% (717)	10.82	(5.46, 21.43)	<.0001
Signs and symptoms[a]					
None or mild	18.7% (140)	81.3% (610)	(Ref)	(Ref)	(Ref)
Moderate	12.9% (90)	87.1% (610)	2.36	(1.59, 3.50)	<.0001
Severe	6.2% (34)	93.8% (516)	5.74	(3.18, 10.35)	<.0001
Comorbid conditions[a]					
None or mild	15.3% (92)	84.7% (508)	(Ref)	(Ref)	(Ref)
Moderate	15.7% (118)	84.3% (632)	1.49*	(1.06, 2.08)	0.0219
Severe	8.3% (54)	91.7% (596)	4.11*	(2.82, 5.99)	<.0001
Quality of Life					
No or minimal impairment	11.2% (84)	88.8% (666)	(Ref)	(Ref)	(Ref)
Mild to moderate impairment	17.2% (129)	82.8% (621)	1.09*	(0.75, 1.57)	0.6500
Significant impairment	10.2% (51)	89.8% (449)	1.83*	(1.09, 3.05)	0.0215

Abbreviations: M-DA – Mild Disease Activity; S-DA – Severe Disease Activity, OR – odds ratio, Ref – Reference level.

An OR above 1 indicates that participants were more likely to choose more severe disease activity when the parameter level of interest was shown.

[a]The survey used patient-friendly language which described “comorbid conditions” as “related conditions” and “signs and symptoms” as “symptoms”. *OR significantly different from the IGF-I disease parameter for the same attribute level

Table B-2 Single-profile choice task overall frequency and output of multivariable model assessing choices for [Mild Disease Activity] vs [Significant Disease Activity]

Disease parameter	Participant Assessment of presence of disease activity: M-DA vs S-DA				
	Frequency of selection		Multivariable model (M-DA is reference outcome)		
	M-DA	S-DA	OR	95% CI	P-value
IGF-I					
Normal	54.2% (243)	45.8% (205)	(Ref)	(Ref)	(Ref)
Slightly elevated	52.9% (360)	47.1% (320)	1.62	(1.25, 2.10)	0.0003
Significantly elevated	35.7% (217)	64.3% (391)	5.68	(3.68, 8.78)	<.0001
Tumor size					
No change	61.5% (302)	38.5% (189)	(Ref)	(Ref)	(Ref)
Slight increase	59.1% (312)	40.9% (216)	1.55	(1.17, 2.06)	0.0025
Significant increase	28.7% (206)	71.3% (511)	10.19*	(6.60, 15.75)	<.0001
Signs and symptoms[a]					
None or mild	47.5% (290)	52.5% (320)	(Ref)	(Ref)	(Ref)
Moderate	55.1% (336)	44.9% (274)	1.42	(1.04, 1.95)	0.0294
Severe	37.6% (194)	62.4% (322)	3.88	(2.81, 5.38)	<.0001
Comorbid conditions[a]					
None or mild	58.7% (298)	41.3% (210)	(Ref)	(Ref)	(Ref)
Moderate	43.2% (273)	56.8% (359)	1.21	(0.92, 1.58)	0.1719
Severe	41.8% (249)	58.2% (347)	2.71*	(1.99, 3.70)	<.0001
Quality of Life					
No or minimal impairment	56.9% (379)	43.1% (287)	(Ref)	(Ref)	(Ref)
Mild to moderate impairment	45.1% (280)	54.9% (341)	1.23	(0.98, 1.55)	0.0761
Significant impairment	35.9% (161)	64.1% (288)	2.65*	(1.95, 3.59)	<.0001

Abbreviations: M-DA – Mild Disease Activity; S-DA – Severe Disease Activity, OR – odds ratio, Ref – Reference level.

An OR above 1 indicates that participants were more likely to choose more severe disease activity when the parameter level of interest was shown.

[a]The survey used patient-friendly language which described “comorbid conditions” as “related conditions” and “signs and symptoms” as “symptoms”. *OR significantly different from the IGF-I disease parameter for the same attribute level

Table B-3 Double-profile DCE choice results for the conditional logistic model

	Conditional logistic model		
	OR	CI	P-value
IGF-I levels (reference: normal range)			
Slightly elevated	0.74	(0.63, 0.87)	<0.01
Significantly elevated	0.30	(0.23, 0.39)	<0.01
Tumor size (reference: no change)			
Slight increase	0.73	(0.63, 0.86)	<0.01
Significant increase	0.29	(0.22, 0.40)	<0.01
Signs and symptoms (reference: none or mild) [a]			
Moderate symptoms	0.64	(0.55, 0.75)	<0.01
Severe symptoms	0.36	(0.28, 0.47)	<0.01
Comorbid conditions (reference: normal) [a]			
Moderate	0.95*	(0.82, 1.09)	0.42
Severe	0.48*	(0.38, 0.60)	<0.01
Quality of Life (reference: no or mild impairment)			
Moderate impairment	0.62	(0.53, 0.73)	<0.01
Significant impairment	0.32	(0.25, 0.40)	<0.01

Abbreviations: CI – 95% confidence interval; IGF-I – insulin-like growth factor 1; QoL – quality of life

An OR below 1 indicates that participants were less likely to choose a patient profile as representing the better health stat than when the parameter level of interest was shown compared to the reference level of that parameter.

[a] The survey used patient-friendly language which described "comorbid conditions" as "related conditions" and "signs and symptoms" as "symptoms".

*OR significantly different from the IGF-I Disease parameter for the same attribute level.
